# Awareness and Attitude About Ototoxic Drugs Among Medical Doctors in Arar City, Saudi Arabia

**DOI:** 10.7759/cureus.60429

**Published:** 2024-05-16

**Authors:** Ekramy Elmorsy, Dhaidan M Alshammari, Madhawi A Alanazi, Khulud Hamed S Alshammari, Reem S Alanazi, Reem Mohammed Z Alanazi, Shmoukh Mushref Alruwaili, Ghadah khalid H Alanazi

**Affiliations:** 1 Pathology, Northern Border University, Arar, SAU; 2 Otorhinolaryngology, Ministry of Health, Arar, SAU; 3 College of Medicine, Northern Border University, Arar, SAU; 4 Internal Medicine, Northern Border University, Arar, SAU

**Keywords:** arar, salicylates, aminoglycosides, ototoxicity, awareness

## Abstract

Objectives: The purpose of this study was to assess the awareness of ototoxicity among medical doctors in Arar City, Saudi Arabia.

Methods: This is a cross-sectional study based on a pre-formed validated questionnaire (Appendix) that included three sections covering participants’ demographic data (three questions), their attitudes (five questions), and knowledge (13 questions) regarding drug-induced ototoxicity.

Results: After obtaining their informed consent, 213 physicians from government and private sector health facilities in Arar were enrolled in the study. Interns and general practitioners represented 57.8% of the participants; consultants represented 17.8%. Only 71.8% of participants were interested in drug-induced ototoxicity, while 26.3% considered ototoxicity a rare complication. Approximately 90% of the participants were knowledgeable about the adverse effects of drugs on the vestibulocochlear system, and 26.7% reported having experienced cases of drug-induced ototoxicity in their practice. Participants showed an overall knowledge score about ototoxicity of 9.3±3.27 (out of 14). The knowledge score was significantly higher (p-value=0.0007) for participants with more years of clinical experience. The most widely known ototoxic drug for participants was frusemide (72.3%), followed by aminoglycoside (68.5%), while acetaminophen (44.1%) ototoxicity was the least known among participants.

Conclusion: Awareness of drug-induced ototoxicity is satisfactory among physicians in the Northern Borders region. However, workshops about all types of drugs with ototoxic effects and the main lines for the management of drug-induced ototoxicity are recommended to increase awareness.

## Introduction

Hearing loss is a prevalent condition that affects millions of people worldwide and is estimated to be the fourth leading cause of disability globally [1]. This life-altering disability impacts individuals, families, and communities. Hearing loss is estimated to affect over 5% of the global population, and by 2050, the number of people affected is expected to exceed 700 million [2]. To date, the published data do not show the global prevalence of drug-induced ototoxicity, with more interest among researchers in addressing the risk of ototoxicity with each type of suspected ototoxic drug [1-7].

Hearing impairment has diverse etiologies, ranging from genetic factors to exposure to ototoxic substances, which are known to cause functional impairment and cellular damage to the tissues of the inner ear [3]. Medications such as aminoglycoside antibiotics, cisplatin, loop diuretics, and nonsteroidal anti-inflammatory drugs (NSAIDs) and exposure to environmental toxins such as heavy metals and solvents have been identified as ototoxic agents via different suggested mechanisms, including mitochondrial bioenergetic disruption and oxidative damage to the vestibulocochlear system [4]. Macrolides, such as erythromycin and azithromycin, showed ototoxic effects if administered intravenously in high doses [5,6]. Glycopeptides, such as vancomycin and teicoplanin, are ototoxic in patients with reported renal impairment [7]. In addition, ototoxicants such as aminoglycosides and platinum-based chemotherapeutics can also cause kidney damage and associated renal dysfunction [1].

Platinum-derived chemotherapies, such as cisplatin, carboplatin, and oxaliplatin, are potent ototoxicants with reported bilateral, progressive, irreversible, and dose-dependent sensorineural hearing loss as soon as the first administration or within several months after the treatment course, with a high need for hearing monitoring before, during, and after the prescribed treatment courses. The risk of hearing loss in patients treated with cisplatin ranges from 10% to 90% for multiple administrations and nearly 30% for a single dose [8,9]. Other ototoxic drugs include high doses of furosemide and other loop diuretics for prolonged periods, which may cause temporary or permanent deafness [10,11]. With prolonged administration, antimalarial drugs such as quinine, chloroquine, and NSAIDs can cause hearing impairment and generally reversible and dose-dependent tinnitus [12,13].

Ototoxic drugs can lead to observable changes in the anatomy and electrophysiology of the auditory system. Symptoms range from temporary tinnitus to permanent deafness and/or mild imbalance to total incapacitation [14]. Much less frequently, these ototoxins can affect central auditory pathways. This complicated neural pathway, which is primarily engaged in complex pattern analysis of acoustic signals, is highly vulnerable to neurotoxicity. Therefore, ototoxicants are also considered neurotoxic [4].

In the context of medical practice, the decisions made by doctors regarding the use of potentially ototoxic medications are crucial. Such decisions depend on their knowledge of ototoxicity, their attitudes toward it, and the available treatment options. As no studies have been done in Arar, Saudi Arabia, regarding medical doctors’ awareness of ototoxicity, this study aims to fill that gap. Understanding their level of awareness is essential for preventing hearing loss and improving patient care.

## Materials and methods

The current study design was approved by the local bioethics committee of Northern Border University (approval decision 20/24/H). The cross-sectional study included medical doctors currently practicing in healthcare facilities within Arar, Saudi Arabia, with the exclusion of any responses received from physicians who are working outside Arar.

Sampling tool: A version of the questionnaire used by Bora et al. (2020) [15] and Tobih et al. (2019) [16] was used with modifications and translated into Arabic by the researchers. The translation was validated via a pilot study that included 20 volunteers. Their responses were not included in the final data results. Survey internal consistency was checked using Cronbach’s alpha with an estimated value of 0.91 indicating strong consistency.

The electronic form contained informed consent discussing the details of the scope of the study before beginning the questionnaire, which was composed of four sections. The first section contained three questions about the participants’ demographic data, including their workplace, professional classification, and work experience. The second section included five questions covering the attitudes and opinions of the participants regarding the incidence and seriousness of drug-induced ototoxicity and their attitudes about informing patients of the expected ototoxic effects of some prescribed medications. The third section comprised 13 questions regarding participants’ knowledge of ototoxic drugs, including the proper protocols to deal with their induced ototoxic effects. The last section included one question regarding the participants’ primary source of data about drug-induced ototoxicity.

Data were collected through online questionnaires. The sample size was identified using the equation n = z2p (1-p)/e2 (n = sample size, z = degree of confidence based on the standard normal distribution, p = approximate proportion of the population exhibiting the trait, and e = tolerated margin of error). The sample size for robust data was expected to comprise more than 200 participants from various health facilities. Participants were selected randomly from the entire population of doctors in Arar who agreed to complete the questionnaire.

To evaluate the participants’ knowledge, questions with right answers were given a score of “one,” while wrong answers and unsure responses were given a score of “zero.”

Statistical analysis: Data were collected in a Microsoft Excel file (Microsoft Office 365) and then analyzed using GraphPad Prism 5 (GraphPad Software Inc., San Diego, CA, USA). Column statistics and the chi-square test were used for data analysis, with a p-value <0.05 considered statistically significant.

## Results

This study aims to assess the awareness of ototoxicity among medical doctors in Arar, Saudi Arabia. Understanding their level of awareness is essential for preventing hearing loss and improving patient care. In total, 213 physicians from the different government and private sector health facilities in Arar were enrolled in the study (Table 1).

**Table 1:** Enrolled participants demographic data

Parameter	Variable	Number	%
Workplace	Eradah Complex and Mental Health, Northern Border Region	3	1.4
	Maternity and Children Hospital (MCH)	22	10.3
	Northern Border University (NBU)	96	45.1
	Primary Health Care Centers (PHCC)	32	15.0
	Prince Abdulaziz bin Musaed Hospital (PAMH)	37	17.4
	Private sector	23	10.8
Saudi Commission for Health Specialties (SCFHS) classification	Consultant	38	17.8
	General practitioner GP	62	29.1
	Intern	59	27.7
	Registrar	22	10.3
	Resident	13	6.1
	Senior registrar	19	8.9
Work experience. (years)	< 1year	85	39.9
	1-5	54	25.4
	6-10	25	11.7
	11-20	33	15.5
	>20	16	7.5
Total		213	100

Attitude question responses showed that 71.8% were interested in drug-induced ototoxicity, while around 85% of the enrolled physicians showed their interest in informing their patients about the suspected ototoxic effect of the prescribed medications. Around one-fourth of the physicians had reported their own practice experience with cases of iatrogenic ototoxicity. Interestingly, around 72% of physicians support the idea that all over-the-counter (OTC) drugs that are ototoxic should be changed to prescription drugs (Rx) to prevent unknown consumption of ototoxic drugs that might lead to hearing loss (Table 2).

**Table 2:** Participants responses to the questionnaire attitude question about drug-induced ototoxicity

Questions	Yes		No		Unsure	
	n	%	n	%	n	%
I am interested in the ototoxic effect of the drugs.	153	71.8	29	13.6	31	14.6
I am interested in informing the patient about the expected ototoxic effect.	181	85.0	13	6.1	19	8.9
I believe ototoxicity is rare from prescribed drugs.	122	57.3	41	19.2	50	23.5
I have experienced cases of ototoxicity in my practice.	56	26.3	137	64.3	20	9.4
All over-the-counter (OTC) drugs which are ototoxic should be changed to prescription drugs (Rx) to prevent unknown consumption of ototoxic drugs that might lead to hearing loss.	154	72.3	22	10.3	37	17.4

Regarding the knowledge section, data revealed that around 90% of the participants were knowledgeable regarding the adverse effects of drugs on the vestibulocochlear system. While only around 40% of the participants were aware of its big numbers among the used medications on the market (around 200 preparations). Around 57% were aware of the importance of baseline audiometry before starting the courses of ototoxic drugs, while 80% were aware of audiologist consultation with suspected cases of drug-induced ototoxicity. The most commonly known ototoxic drug for participants was frusemide (72.3%), followed by aminoglycosides (68.5%) and acetaminophen (44.1%), whose ototoxicity was the least known among participants (Table 3).

**Table 3:** Participants responses to the questionnaire knowledge question about drug-induced ototoxicity

Question	Yes		No		Unsure		Right answers	
	n	%	n	%	n	%	n	%
Some drugs can cause hearing impairment.	195	91.5	5	2.3	13	6.1	195	91.5
Certain medications can damage the ear, resulting in a ringing sensation in the ear.	190	89.2	8	3.8	15	7.0	190	89.2
Some drugs may cause balance disorders and vertigo.	196	92.0	7	3.3	10	4.7	196	92.0
I'm aware that there are more than 200 ototoxic drugs sold by pharmaceutical companies in the market.	89	41.8	38	17.8	86	40.4	89	41.8
The patients who are prescribed ototoxic drugs are referred for a baseline record of hearing and balance to be recorded by an audiologist.	122	57.3	42	19.7	49	23.0	122	57.3
I will refer patients to an audiologist in cases with expected cases of ototoxicity.	171	80.3	23	10.8	19	8.9	171	80.3
For cases in which the medications have already been taken and cannot be stopped or changed, the patient and the audiologist can take steps to manage the effects of the hearing loss that results.	153	71.8	15	7.0	45	21.1	153	71.8
Aminoglycoside antibiotics, such as gentamicin, are medications known to cause permanent hearing loss.	146	68.5	19	8.9	48	22.5	146	68.5
Furosemide may have ototoxic effects.	154	72.3	23	10.8	36	16.9	154	72.3
Non-steroidal anti-inflammatory drugs such as salicylates may have ototoxic effects.	125	58.7	26	12.2	62	29.1	125	58.7
loop diuretics such as furosemide are known to cause temporary hearing loss.	110	51.6	20	9.4	83	39.0	110	51.6
Antimalarial quinine, is known to cause temporary hearing loss.	138	64.8	12	5.6	63	29.6	138	64.8
Cancer chemotherapy drugs, such as cisplatin and carboplatin, are known to cause permanent hearing loss.	122	57.3	11	5.2	80	37.6	122	57.3
Acetaminophen was reported to have ototoxicity.	94	44.1	37	17.4	82	38.5	94	44.1

Generally, participants showed an overall knowledge score about ototoxicity of 9.3±3.27 (out of 14). The knowledge score was significantly higher (p-value = 0.0007) with more clinical experience (Table 4). 

**Table 4:** The effect of the participants’ Saudi Commission for Health specialty classification and their years of work experience on the knowledge score concerning drug-induced ototoxicity. ** means p-value <0.001

Parameter	Variable	Knowledge score		One way ANOVA p-value
		Average	SD	
Saudi Commission for Health Specialties (SCFHS) classification	Consultant	9.737	2.648	0.47
	General practitioner (GP)	9.71	3.281	
	Intern	8.797	3.516	
	Registrar	9.682	3.077	
	Resident	8.462	4.274	
	Senior registrar	9	2.103	
Work experience (years)	< 1year	8.353	3.591	0.0007**
	1–5	9.5	3.189	
	6–10	10.21	2.57	
	11–20	10.42	2.705	
	>20	11	2.782	
Average		9.3	3.27	

Interestingly, participants reported that undergraduate and postgraduate studies were the main source of their knowledge about ototoxic drugs, with a limited role in continuous medical education programs, learning, and training activities (only 22%% of participants).

## Discussion

The current study evaluated awareness and attitudes among physicians in Arar about ototoxicity and ototoxic agents. After securing their informed consent, 213 physicians from government and private sector health facilities in Arar were enrolled. Interns and general practitioners represented 57.8% of the participants; consultants represented 17.8%. Of the participants, 71.8% expressed interest in ototoxicity, and 26.3% considered it a rare complication. Around 90% of the participants were knowledgeable regarding some drugs’ adverse effects on the vestibulocochlear system, and 26.7% reported experiencing cases of drug-induced ototoxicity in their practice. Participants demonstrated an overall knowledge score about ototoxicity of 9.3±3.27 (out of 14). The knowledge score was significantly higher (p-value=0.0007) among participants with more years of clinical experience. The ototoxic drug most widely known among the study participants was frusemide (72.3%), followed by aminoglycoside (68.5%); acetaminophen (44.1%) ototoxicity was the least known among participants.

The current study data revealed that participants showed interest in ototoxicity at 71.8%, while 26.3% considered it a rare complication. Around 90% of the participants were knowledgeable about the adverse effects of drugs on the vestibulocochlear system, and 26.7% reported experiencing cases of drug-induced ototoxicity in their practice. There was a high level of awareness (approximately 90%) of the effects of ototoxicity, including hearing loss, tinnitus, and balance disorders. These data were within the range of Tobih et al.’s (2019) study [16] from Nigeria. They showed a higher level of awareness than that reported by Bora et al. (2020) [15] from India, who reported awareness of approximately 80% for drug-induced hearing loss and tinnitus and around 60% for balance disorders. Of the participants, 85% thought it necessary to inform patients about the risk of ototoxicity when prescribing a medication with a potential ototoxic effect. This may be explained by their belief that ototoxicity is a rare complication, along with other compelling factors, such as the possibility of disclosing the risk of side effects that may affect the patient’s compliance with the prescription. Further, they may believe in the efficacy of these ototoxic drugs and that their benefits outweigh the risks. As a result, only 57.3% of the participants were convinced they should request an audiogram before initiating a treatment course using a drug with potential ototoxic effects. Lastly, 80.3% were aware of the need to refer patients to an audiologist for consultation in suspected cases of drug-induced ototoxicity.

The current data revealed that the most widely known ototoxic drug for participants was frusemide (72.3%), followed by aminoglycoside (68.5%); acetaminophen (44.1%) ototoxicity was the least known among participants. Furosemide is well known to cause temporary hearing loss, with rare reports of permanent deafness, when used in cases of severe acute or chronic renal failure or in combination with other ototoxic medications. It causes edematous spaces in the epithelium of the stria vascularis of the cochlea due to altered Na+/K+-ATPase and Na-K-2Cl channels, with decreased endolymphatic potential and loss of normal cochlear microphonic, summating, and action potentials [10,17]. Aminoglycoside ototoxicity is especially common among patients with tuberculosis who are receiving streptomycin therapy [18]. Aminoglycosides cause cochlear sensory cell degeneration, which initially spreads from the basal turn to the cochlea apex with earlier high-frequency hearing loss. Vestibular impairment is manifested by ataxia and nystagmus due to vestibular sensory cell damage in the crista ampullaris [19]. Aminoglycosides such as streptomycin and gentamycin are predominantly vestibulotoxic, whereas neomycin and kanamycin are more toxic to the cochlea [20]. Paracetamol is a widely used OTC drug with an overlooked potential for ototoxicity [21]. Interestingly, around 70% of participants support the concept that all OTC drugs that are ototoxic should be changed to Rx to prevent unknown consumption of ototoxic drugs, which could lead to hearing loss.

The current study was conducted via an online survey with the following advantages: low cost, increased response rates, and convenience. On the other hand, the data have the limitations of online survey-based studies, such as biased samples with fake answers, sampling, and respondent access issues. However, online surveys are a widely accepted and rapidly expanding method for cross-sectional survey data collection.

## Conclusions

The current study has shown that there is a high level of awareness of ototoxic drugs among healthcare professionals. However, fewer are willing to inform patients about the risk of ototoxicity, and an even lower percentage are interested in requesting a baseline audiogram before prescribing ototoxic drugs. Despite this awareness, ototoxic drugs are widely used, which may be because they are relatively cheaper, widely available, and can be taken for shorter durations. This necessitates an urgent, aggressive public health campaign and government strategic policy intervention to address the issue of adequate provision of alternative drugs with concurrently favorable costs.

## Appendices

Questionnaire in English

**Table 5:** Section A: Demographic data

Question		Choice’s list
1	Workplace:	Eradah Complex and Mental Health – Northern Border regoin
		Maternity and Children Hospital (MCH)
		Northern Border University (NBU)
		Primary health care Center (PHCC)
		Prince Abdulaziz bin Musaed Hospital (PAMH)
		Private sector
2	Saudi Commission for Health Specialties classification:	Consultant
		Senior registrar
		Registrar
		Resident
		Interns
3	Work experience:	Less than 1 year
		1–5 years
		6–10 years
		11–20 years
		More than 20 years

**Table 6:** Section B: Attitude about drugs induced ototoxicity

Questions		Answers		
		Yes	No	Unsure
1	I am interested in the ototoxic effect of the drugs.			
2	I am interested in informing the patient about the expected ototoxic effect.			
3	I believe ototoxicity is rare from prescribed drugs.			
4	I have experienced cases of ototoxicity in my practice.			
5	All over-the-counter (OTC) drugs which are ototoxic should be changed to prescription drugs (Rx) to prevent unknown consumption of ototoxic drugs that might lead to hearing loss.			

**Table 7:** Section C: Knowledge about drugs induced ototoxicity

Questions		Answers		
		Yes	No	Unsure
1	Some drugs can cause hearing impairment.			
2	Certain medications can damage the ear, resulting in a ringing sensation in the ear.			
3	Some drugs may cause balance disorders and vertigo.			
4	I’m aware that there are more than 200 ototoxic drugs sold by pharmaceutical companies in the market.			
5	Aminoglycoside antibiotics, such as gentamicin, are medications known to cause permanent hearing loss.			
6	Furosemide may have ototoxic effects.			
7	Non-steroidal anti-inflammatory drugs such as salicylates may have ototoxic effects.			
8	Antimalarial quinine is known to cause temporary hearing loss.			
9	Cancer chemotherapy drugs, such as cisplatin and carboplatin, are known to cause permanent hearing loss.			
10	Acetaminophen was reported to have ototoxicity.			
11	The patients who are prescribed ototoxic drugs are referred for a baseline record of hearing and balance to be recorded by an audiologist.			
12	I will refer patients to an audiologist in cases with expected cases of ototoxicity.			
13	For cases in which the medications have already been taken and cannot be stopped or changed, the patient and the audiologist can take steps to manage the effects of the hearing loss that results.			

**Table 8:** Section D: Data Sources

Question		Choice’s list
1	My source of data about ototoxic agents is/are:	My undergraduate studies
		My postgraduate studies
		Workshops, conferences, and continuous medical education (CME) activities
